# A Role for Fragment-Based Drug Design in Developing Novel Lead Compounds for Central Nervous System Targets

**DOI:** 10.3389/fneur.2015.00197

**Published:** 2015-09-11

**Authors:** Michael J. Wasko, Kendy A. Pellegrene, Jeffry D. Madura, Christopher K. Surratt

**Affiliations:** ^1^Mylan School of Pharmacy, Graduate School of Pharmaceutical Sciences, Duquesne University, Pittsburgh, PA, USA; ^2^Department of Chemistry and Biochemistry, Center for Computational Sciences, Bayer School of Natural and Environmental Sciences, Duquesne University, Pittsburgh, PA, USA

**Keywords:** fragment-based drug design, CNS, dopamine, structure, target

## Abstract

Hundreds of millions of U.S. dollars are invested in the research and development of a single drug. Lead compound development is an area ripe for new design strategies. Therapeutic lead candidates have been traditionally found using high-throughput *in vitro* pharmacological screening, a costly method for assaying thousands of compounds. This approach has recently been augmented by virtual screening (VS), which employs computer models of the target protein to narrow the search for possible leads. A variant of VS is fragment-based drug design (FBDD), an emerging *in silico* lead discovery method that introduces low-molecular weight fragments, rather than intact compounds, into the binding pocket of the receptor model. These fragments serve as starting points for “growing” the lead candidate. Current efforts in virtual FBDD within central nervous system (CNS) targets are reviewed, as is a recent rule-based optimization strategy in which new molecules are generated within a 3D receptor-binding pocket using the fragment as a scaffold. This process not only places special emphasis on creating synthesizable molecules but also exposes computational questions worth addressing. Fragment-based methods provide a viable, relatively low-cost alternative for therapeutic lead discovery and optimization that can be applied to CNS targets to augment current design strategies.

## Introduction

The cost of developing and bringing a single successful drug to market approaches one billion dollars, and the process requires on average 12 years to accomplish. Even after FDA approval, only one in five medications is eventually profitable (1). The preclinical evaluation process is estimated to be 32% of the total cost of drug design (2). The recent economic recession forced pharmaceutical companies to drastically limit research expenses, and while outsourcing is an option carrying benefits and liabilities (3), development of new, more cost-effective drug design methods is a priority. Central nervous system (CNS) disorders are logical foci for such new strategies; the increasingly geriatric population is more susceptible to Alzheimer’s disease, Parkinson’s disease, and ischemic stroke (4). Mental health disorders such as depression are effectively treated with existing therapeutics only a fraction of the time; much of the population is unresponsive or plagued with adverse drug effects (5). Among the reviews and discussion on structure- and knowledge-based CNS drug design (4, 6–9), recent fragment-based drug design (FBDD) literature focusing on CNS targets is underrepresented. Application of FBDD to CNS targets should provide a new spark for drug design in this area.

## What is Virtual Drug Design?

**Figure 1:**
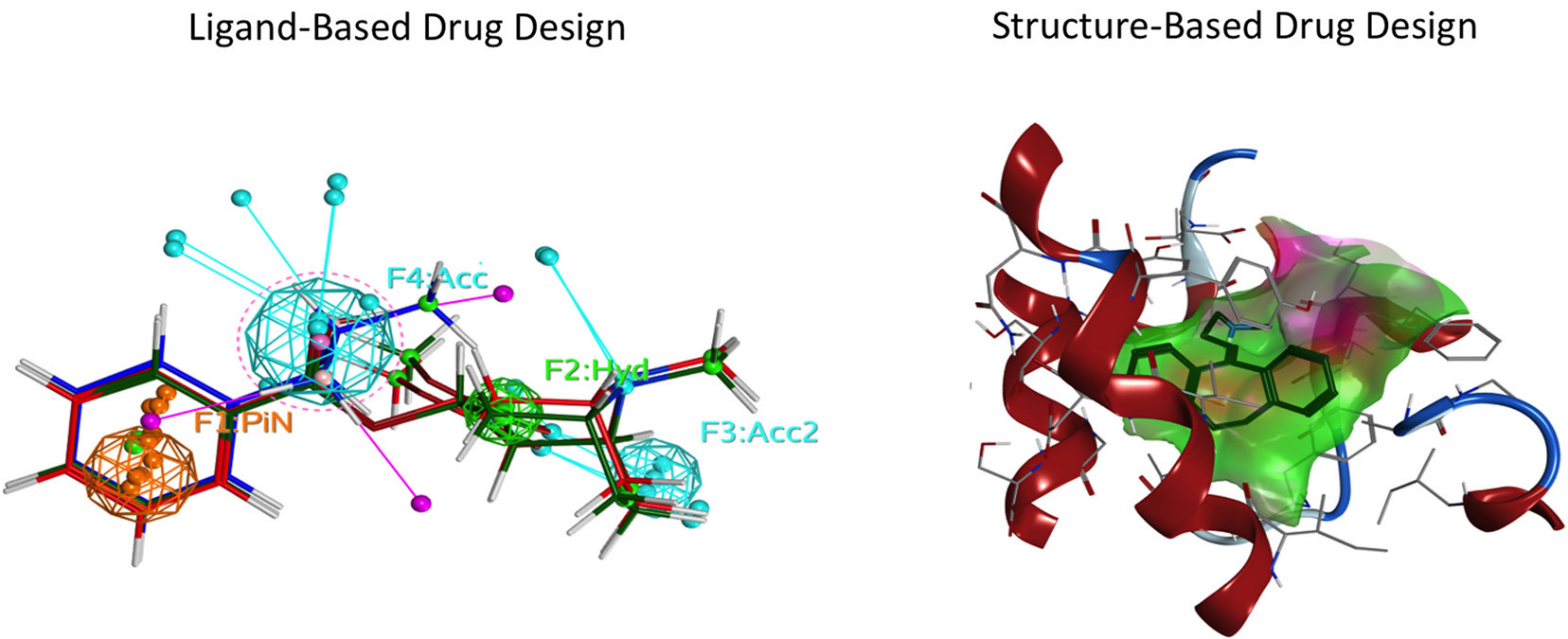
**Representation of two commonly used drug design approaches**. Ligand-based drug design focuses on specific properties of a molecule, employing a pharmacophore. Structure-based drug design utilizes the ligand-binding pocket amino acid side chains of the target receptor. Left: ligand-based drug design. Spheres indicate the features of the ligand pharmacophore, including pi–pi bond (PiN, orange), hydrophobic (Hyd, green), and H-bond acceptor (Acc, cyan) interactions. Right: structure-based drug design. The ligand (blue) is docked in the orthosteric pocket (green cloud) of a G protein-coupled receptor (red).

**Figure 2:**
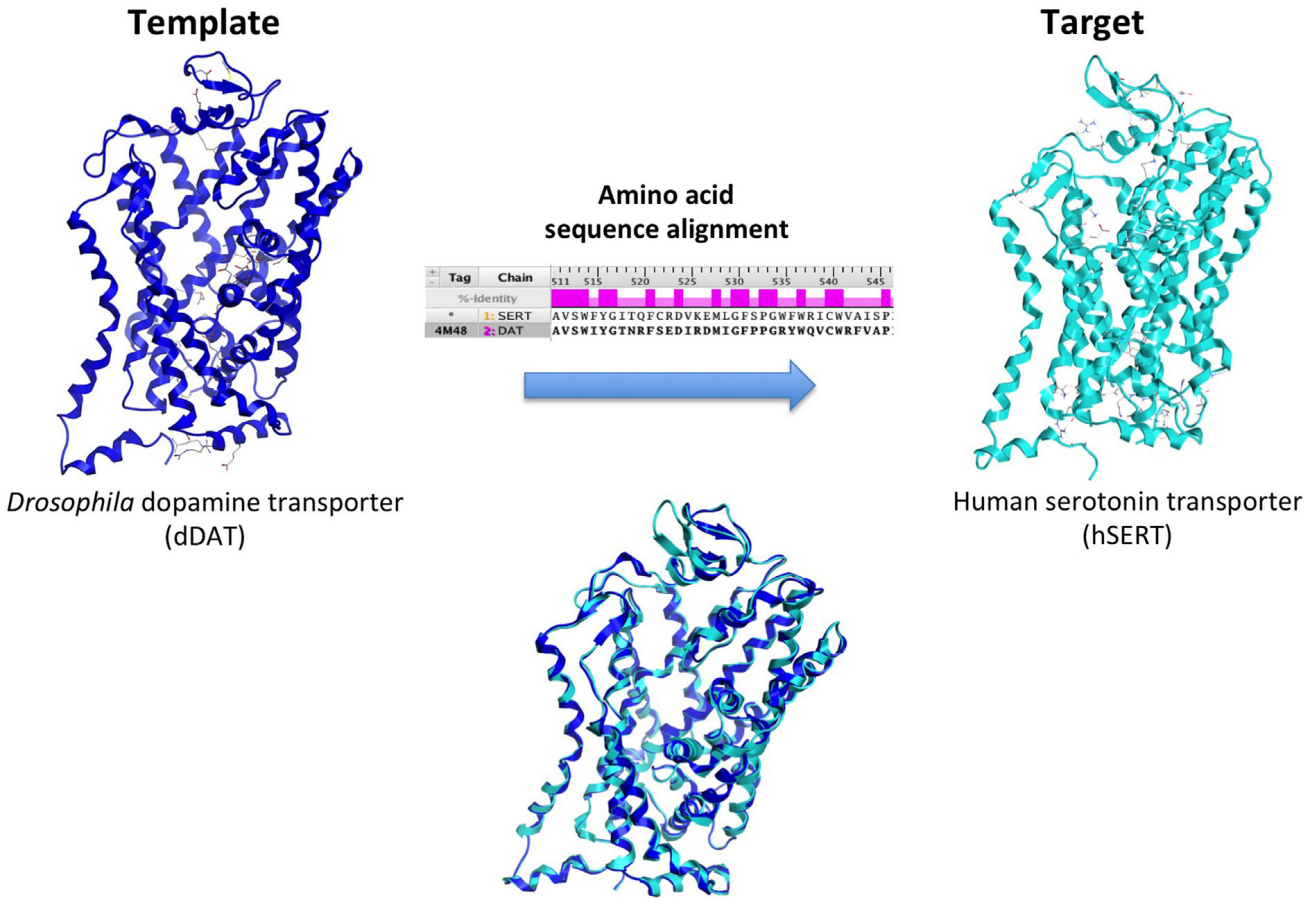
**Creation of a human serotonin transporter (hSERT) homology model**. The SERT primary amino acid sequence is aligned with optimal fit with that of the x-ray crystal structure template, the dopamine transporter (DAT) protein from the fruit fly *Drosophila melanogaster* (dark blue). This alignment, combined with structural information from the template, is used to create a human SERT homology model (cyan).

Ligand- and structure-based techniques are most commonly used in virtual drug design (Figure 1). Ligand-based techniques involve comparing candidate ligands to an experimentally verified ligand for a given receptor, and can be performed without knowledge of the receptor’s structure. When a 3D receptor structure is available, structure-based drug design (SBDD) is an attractive alternative (5). Receptor protein structures are experimentally solved through X-ray crystallography or by NMR techniques (10). If the target protein of interest has not been crystalized, a homology model can be created using as template a crystal structure of an evolutionarily similar protein (Figure 2). Template crystal structures are available for download from the Protein Data Bank^1^ and the Cambridge Structure Database^2^. As the structures of targets represented by homology models are not experimentally verified, one might question how these models compare to experimentally known structures. A retrospective docking study on the β_2_-adrenergic receptor (AR) noted the usefulness of homology modeling even when a crystal structure is known. Crystal structures and homology models based on different templates were compared with respect to various conformational states. The homology models were found to be more useful in differentiating active and inactive compounds and provided more conformational flexibility, increasing the diversity of compounds that could be accommodated by the active site (11).

**Figure 3:**
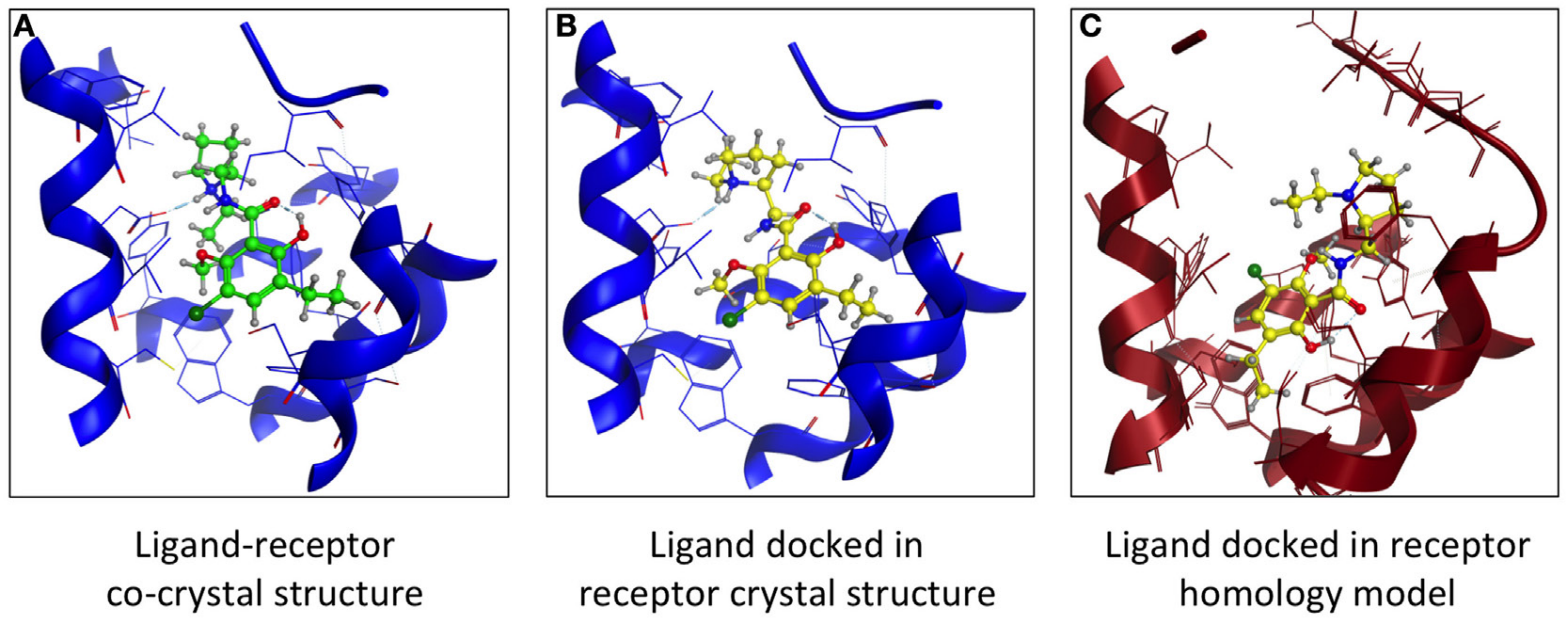
**Comparing the binding of a GPCR orthosteric antagonist at a homology model vs. crystal structure**. **(A)** Structure of the dopamine D3 receptor (blue) – eticlopride (green) crystal structure (13). **(B)** Docking of eticlopride (yellow) into the D3R crystal structure (blue). **(C)** Docking of eticlopride (yellow) into a D3R homology model (red) based on the β_2_-adrenergic receptor crystal structure (60).

Another comparison of structures examined dopamine D3 receptor (D3R) homology models based on β_1_- and β_2_-AR crystal structures. Both models had comparable VS hit rates and showed no bias toward their respective templates (12). Using the D3R-eticlopride cocrystal (13) as template, we created a D3R model lacking the D3 orthosteric antagonist eticlopride. This ligand was docked in the D3R model, and its position was compared to that found in the crystal structure (Figure 3). The location of the docked eticlopride within the model was very similar to its crystallized position (Figures 3A,B). More deviation of the ligand’s original position was observed when the β_2_AR-based D3R model was employed [Figure 3C, based on Ref. (12)]. Nevertheless, a crystal structure is a static representation of a protein and cannot account for the multiple conformational states within the protein–ligand complex (14). While the structural information derived from a crystal structure is useful, it is akin to a “snapshot” and cannot fully represent all conformational states of a protein.

### Lead compound development

A lead compound is the precursor molecule that through modifications becomes the therapeutic drug. The quality of a “lead” is important; substandard leads hinder the discovery process by unnecessarily diverting resources. Good leads increase the likelihood of progressing through later phases of clinical testing, justifying the extra effort spent generating such a compound (1).

High-throughput screening (HTS) is a traditional method of discovering new lead compounds. A chemical library of thousands to millions of compounds is gathered and pharmacologically tested at one or more receptors to identify potential “hits.” The assays may assess the compound’s receptor-binding affinity or functional response (e.g., Ca^2+^ channel opening, vasodilation, analgesia) (15). HTS is an effective process for identifying lead compounds, but the money and time required are typically considerable. Automation is implemented due to the sheer number of compounds being screened (16). The associated cost of HTS is well beyond what the typical academic researcher can afford, and minimally requires a core facility (5).

Virtual screening (VS, also known as virtual HTS or vHTS) is an emerging alternative for discovering new lead compounds. VS uses computational methods to predict how the compounds in a structural (virtual) library would interact with a crystal structure representation or homology model of the target receptor (17). Compounds in a virtual library are ranked by their predicted binding affinities or other criteria (e.g., visual inspection or druggability). The top-scoring hit compounds can be refined *in silico* to improve characteristics, such as binding affinity or lipophilicity, before selecting hits to be pharmacologically evaluated. Thus, VS provides a rapid and inexpensive “pre-filter” step that can reduce the time and cost associated with conventional HTS.

### Guidelines for identifying drug-like molecules

A landmark 1997 paper on identifying drug-like molecules (18) provided guidelines for predicted drug-like properties, now known as the Lipinski’s Rule of Five. Molecules with more than 5 hydrogen bond donors, 10 hydrogen bond acceptors, a molecular weight >500 g/mol, or a LogP value >5 were predicted to show poor solubility and permeability (18). The Rule of Five was designed to be a filter that could be applied to computational methods to predict better starting compounds for drug discovery. These serve more as guidelines rather than inflexible rules; suitable compounds have been found that did not meet every criterion. Still, Christopher Lipinski has stated that Pfizer would not pursue compounds that broke two of the five parameters (19).

## Introduction to Fragment-Based Drug Design

### Fragments in drug design

Fragment-based drug design is a process in which new leads are developed/identified by sequentially piecing together molecules. Fragments are drawn from three sources: known biologically active drugs, natural products, and compounds with novel scaffolds (20). Fragments generally have a molecular weight of <250 Da and a LogP < 3 (21). An important difference between fragments and whole molecules is the typically poor initial binding affinity of the former. The fragments are later “grown” into high-affinity ligands through the drug design processes described below.

### Historically significant fragment-based programs

The use of fragments in rational drug design is a concept originating from the late 1980s (Table 1). Among the original fragment-based approaches is the GRID program. This computational approach to SBDD creates a grid within the receptor’s ligand-binding pocket. The functional group of a probe molecule is placed at each point within the grid and measured for its ability to interact with the pocket. Grid points of equal interaction strength are connected to form a contour map of the binding pocket, which allows easy identification of potential regions of interest to exploit (22). The multiple-copy simultaneous search (MCSS) method was also designed to explore the receptor’s ligand-binding pocket (23). The binding site of hemagglutinin, part of the influenza virus, was initially probed with thousands of fragments simultaneously, followed by energy minimization and/or quenched molecular dynamics. Fragments were composed of three to six atoms with little or no dihedral degrees of freedom, yet were complex enough to model the potential interactions within the binding site. Points of interest within the binding pocket could be identified based on the aggregation of the fragments from the minimization and exploited in rational drug design (23). This method was further detailed in a second paper in 1993 that used MCSS to construct ligands targeting the human immunodeficiency virus 1 proteinase (24). The MCSS method fit into a three-part strategy of drug design: development of a method to identify regions of the binding pocket that interacted favorably with the functional group fragments, linkage of the identified fragments to form novel ligands, and prediction of high-affinity binders among the newly formed structures.

**Table 1:** **Key software advances in FBDD**.

Software	Year	Importance	Paper
GRID	1985	Ligand-binding pocket mapping with probe molecules	(22)
MCSS	1991	Simultaneous search of the binding pocket with probe molecules followed by minimization	(23)
LUDI	1992	Focus on hydrogen bonding and linking of fragments to form inhibitors	(25)
SPROUT	1993	Incorporation of primary and secondary characteristics of the receptor into the ligand design process	(26)
SuperStar	1999	Knowledge-based approach using information from crystal structures	(27)
MUSIC	2000	Simultaneous search minimization method performed within a flexible binding pocket created incorporation of molecular dynamics	(28)

In 1992, an automated process to design enzyme inhibitors (“LUDI”) was described. This program utilizes the ligand-binding site of crystal structures and small molecule probes to identify ligand–receptor interaction sites. The focus is placed on receptor hydrogen bonding ability with the probes; the latter are then replaced with fragments that can be joined to form novel inhibitors (25). SPROUT, introduced in 1993, employs primary and secondary structure characteristics of the receptor to generate ligands. Once these characteristics are identified, a fragment library can be screened to find molecules that match the constraints, which could then be combined to form novel compound scaffolds and ranked for predicted affinity (26).

The late 1990s brought two additional programs, SuperStar and “multi-unit search for interacting conformers” (MUSIC). SuperStar models binding pocket interactions using a knowledge-based approach created by studying ligand interactions in experimentally solved receptor crystal structures in the Cambridge Structural Database. This information is translated into a scatterplot, to be used to predict how fragments will interact with the binding site of the target protein (27). MUSIC improved upon the MCSS program introduced by Miranker and Karplus. While MCSS was developed for use with a fixed binding pocket, MUSIC uses a flexible binding pocket to run the multiple-copy simulations. The flexible binding pocket is prepared using a pharmacophore, after molecular dynamics simulations identify possible conformational changes within the receptor (28).

As FBDD moved into the next century, a “rule of three” for working with fragments emerged from Jhoti and colleagues. Based on their analysis of fragment hits, it was suggested that druggable fragments showed three properties: a molecular weight under 300 Da, <3 hydrogen bond donors, and a LogP < 3 (29). This rule has been accepted by many and incorporated into the construction of commercial fragment libraries, such as ChemBridge and Life Chemicals. Recently, the usefulness of this rule of three has been a subject of debate (30, 31). The Köster et al. study built and tested a fragment library that was not limited to the rule of three. Fifty-five endothiapepsin inhibitor hit compounds were identified, 11 of which were crystalized to discern how the fragments bound to the enzyme. Fragments that did not comply with the rule of three were crystallized more frequently than the rule-compliant fragments, suggesting that this rule of three could exclude promising lead compounds. Another concept that has been reviewed recently is ligand efficiency, which tries to quantify the binding energy contribution of a ligand on a per atom basis (32). Whereas typical drug development overemphasizes drug potency in selecting which compounds should advance, ligand efficiency also takes into account the compound’s molecular size, lipophilicity, shape, hydrogen-bonding properties, and polarity. The ligand efficiency approach is useful in assessing which fragments should comprise the drug; these fragments are unlikely to be detected by affinity or potency measurements.

### Fragment libraries

Fragments are pooled to form a fragment library, used by structure-based VS methods to identify starting points for lead compounds (20). Of the various methods for designing fragment libraries (33), structural diversity of the library is key. Fragments are advantageous in this respect over whole compounds because comparable structural diversity can be achieved with far fewer fragments (33). The movement of whole compounds is also more likely to be sterically hindered within the receptor ligand-binding pocket, while fragments are able to easily maneuver in this “chemical space” to optimize intermolecular interactions (33, 34).

Commercial sources have made their fragment libraries available to screen *in silico*. The “ZINC is not commercial” (ZINC) database, operated by the University of California, is composed of 293 commercially available libraries (35). Alternatively, researchers may develop “in house” libraries customized for screening a given target. Focused libraries, small subsections of molecules that contain desired functional groups or qualities, can be created to screen select targets. Virtual fragment screening techniques can be employed to filter libraries (36). Table 2 lists several libraries that can be used for fragment-based VS. These collections were chosen for their accessibility to be screened by a researcher, rather than collections that could be screened by third party companies. The fragment library used at Vernalis was originally composed of vendor catalogs (37). The original 2004 library was designed to be a general-purpose library that could be screened by a variety of targets. Increased emphasis on chemical diversity was placed on the construction of the library, and a molecular fingerprinting method based on 2D three-point pharmacophores was used to assess the diversity. Many compounds were removed from the original library after screening and quality control, replaced with more complex fragments that carried more desirable characteristics. This evolution was considered an essential process to keep up with new project demands and the availability of new information on the desired targets (37). The 3D Fragment Consortium, a collaboration of non-profit drug discovery groups based in the United Kingdom, argues that traditional fragment libraries often contain limited shape diversity, possibly explaining why some target sites are troublesome for trying to identify hit compounds. The consortium is creating a chemical library consisting of fragments that have “greater three dimensionality” that explore the chemical space, as opposed to planar, rigid fragments. While the increased complexity of 3D compounds could potentially lower hit compound numbers, the group is hopeful that the resulting leads will explore more biologically relevant chemical space and lead to better starting molecules for drug design (38).

**Table 2:** **Comparison of VS fragment libraries**.

Fragment library	Key feature	Website
3D Fragment Library Consortium	Greater shape diversity	http://www.3dfrag.org
AnalytiCon	Fragments from natural products	http://www.ac-discovery.com
Asinex	Minimalistic fragments	http://www.asinex.com
ChemBridge	Over 6000 compounds, rule of 3 (RO3)	http://www.chembridge.com/
ChemDiv	Over 14,000 fragments	http://www.chemdiv.com
Enamine	RO3	http://www.enamine.net
Key Organics	Multiple fragment libraries	http://www.keyorganics.net
Life Chemicals	Multiple fragment libraries	http://www.lifechemicals.com
Maybridge	RO3	http://www.maybridge.com
Otava	RO3	http://www.otavachemicals.com
Prestwick Chemical	Fragments derived from known drugs	http://www.prestwickchemical.com
Vitas-M Labs	RO3	http://www.vitasmlab.com
ZINC	Combination of various commercial libraries	http://zinc.docking.org

### Recent FBDD strategies

**Figure 4:**
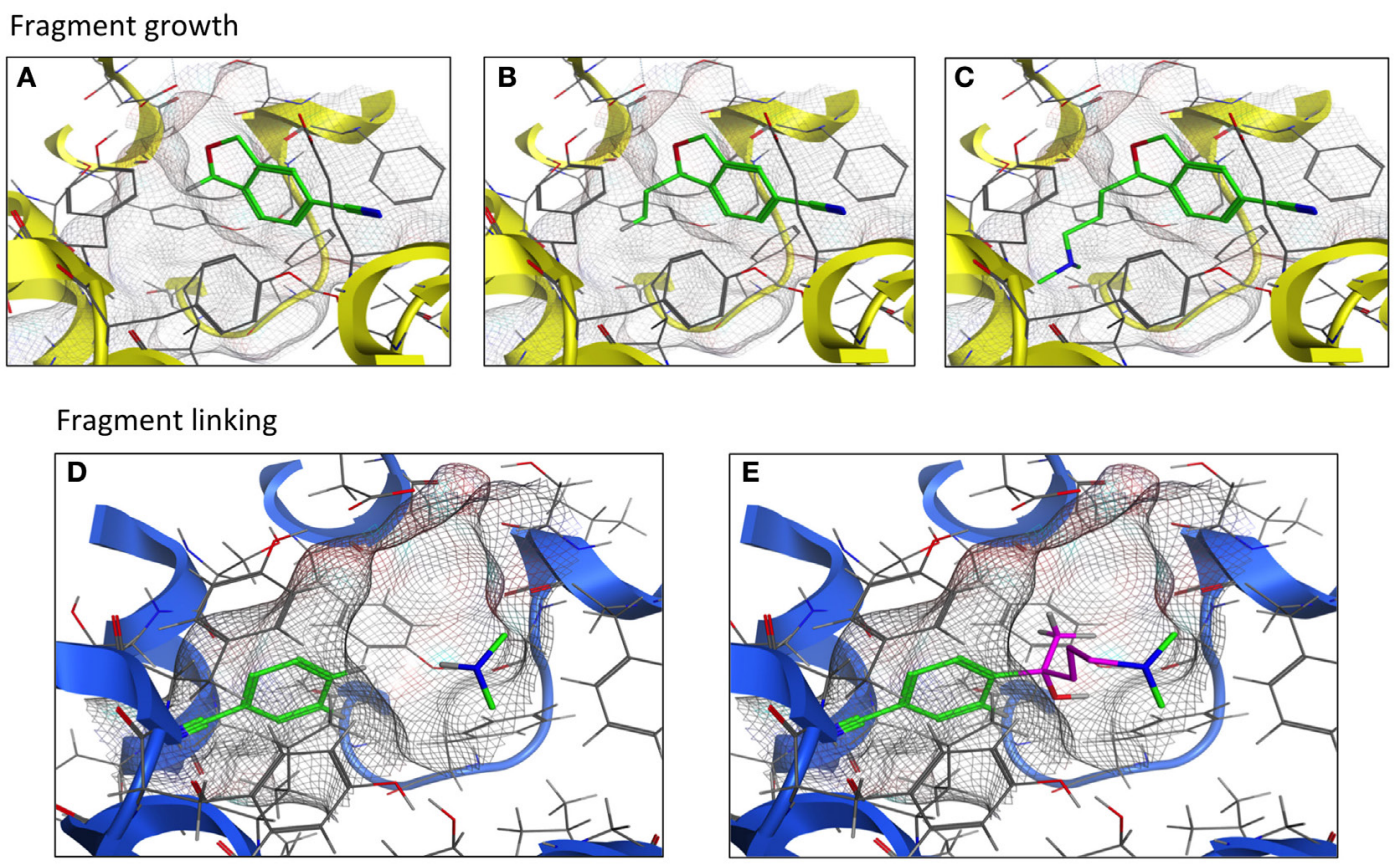
**Fragment growth vs. fragment linking**. Fragment growth **(A–C)** extends the fragment (green) to maximize interactions within the binding pocket (TM helices in yellow). Fragment linking **(D,E)** covalently joins (purple) two or more fragments (green) to form a novel-scaffold ligand within the binding pocket (TM helices in blue).

Techniques and strategies utilized in the development of the fragment into a lead compound are constantly evolving as new concepts are explored and modified. As the name implies, the fragment growing strategy is to start with a fragment within the receptor’s ligand-binding pocket and allow the fragment to expand to interact with the pocket amino acid side chains. A second strategy, fragment linking/merging, first positions fragments to optimally interact with the pocket. These fragments are next covalently joined with “filler” atoms or molecules to form a single molecule that likely provides a novel chemical scaffold (Figure 4). These fragment-based techniques have been applied to CNS targets in the pursuit of structure-based lead design. The initial step is fragment screening to choose the proper starting point; this technique is often referred to as “docking.” VS of a fragment library using a computational model of the histamine H_1_ receptor crystal structure (39) yielded docking fragments in one of the first studies of this nature involving a G protein-coupled receptor (GPCR). The fragment docking was assessed with a fingerprint scoring method that predicted 19 out of 26 fragment-like compounds to possess high binding affinity at the H1 receptor (73% hit rate) (39). Separate work compared fragment library screening by two GPCR models corresponding to the dopamine D_3_ and histamine H_4_ receptors. Molecular dynamics was performed to represent the different conformational states of the receptor-binding pocket. All 12,905 fragments were docked into both a single receptor conformation and an ensemble of conformations. The top 50 hit compounds for each receptor model were pharmacologically tested. Both the single and ensemble structures were found to be suitable for screening against GPCRs; little overlap was observed between the leads from the two receptors (36).

When possible, pharmacological data are used to augment the fragment screening process. The orthosteric pocket of an A_2A_ adenosine receptor model was used to screen a fragment library; 500 fragments were ranked using target-immobilized NMR screening, yielding 94 hits to be pharmacologically characterized. Five fragments were pharmacologically identified to exceed the threshold affinity (30% displacement of a radioligand at 500 μM) for the target. Four of those fragments were among the top 50 fragments predicted *in silico*. While the computational method found most of the pharmacologically relevant fragments, it also predicted similarly high binding affinities for 46 “non-hit” compounds. A secondary screen of the A_2A_ receptor using commercially available fragments yielded 22 compounds, 14 of which were subsequently shown via radioligand-binding assay to be A_2A_ adenosine receptor ligands. Molecular dynamics simulations and quantitative structure–activity relationship (QSAR) were used to refine the lead fragments (40). In separate work, consensus scoring methods (similarity fusion and group fusion) were used to retroactively analyze ligand-based VS of over a thousand fragments that were experimentally tested against the histamine H_1_ and H_4_ and serotonin 5-HT_3A_ receptors (two GPCRs and a ligand-gated ion channel). The results from this study showed that one can increase VS enrichments by using both consensus scoring methods. The authors also recommend that similarity fusion and group fusion be used in a prospective ligand-based VS analysis (41).

Target selectivity may be the most daunting challenge of the drug development process. Many protein targets are evolutionarily similar, which increases the probability of off-target responses. One strategy to increase selectivity arose from structure-based VS using histamine H_4_ receptor and 5-HT_3A_ (serotonin) receptor models that yielded a common pool of hit compounds. Because the more complex hit molecules provided more potential groups for interaction within a given receptor’s ligand-binding pocket, increased complexity correlated with compound selectivity. Studying how compounds interact with the binding pocket can determine which interactions are favored. That knowledge could guide target specificity by extending the fragment to favor the target-selective interactions (42).

While single-target selectivity is typically a goal of drug design, there are occasions in which modulating more than one receptor may be appropriate. A novel strategy for designing ligands with affinity for multiple distinct targets was recently outlined. Through a two-step process, LigBuilder 3 generated ligands with affinity for cyclooxygenase-2 (COX-2) and 5-lipoxygenase/leukotriene A_4_ hydrolase (LTA_4_H), enzymes involved in metabolic pathways of inflammation. First, fragments derived from known inhibitors of COX-2 or LTA_4_H were docked into the crystal structure-based models of both receptors using AutoDock 4.0. Fragments that showed binding for both receptors were “grown” in the second step. Emphasis was placed on testing multiple docking conformations because of the difficulty in predicting fragment docking. Compounds chosen for experimental evaluation were selected based on the need for minimum modification, sharing a common framework, and synthesizability. A second round of optimization was used to further refine the compounds for both receptors (43). This method was able to design *de novo* dual target inhibitors with an increased success rate compared to rival strategies, such as linking and fusion, which covalently link fragments that are simultaneously bound to the receptor pocket.

Considering the similarities between GPCR orthosteric (primary) binding pockets among not-so-distant family members, the drug design field has turned its focus to secondary or allosteric binding sites (44). Allosteric or secondary sites are usually composed of the extracellular loops, where sequence homology is low compared to the transmembrane regions that delineate the orthosteric site. One approach to mapping an allosteric/secondary site while generating novel ligands is to determine the span between the primary and secondary sites using “double-headed” molecular probes. Using a dopamine D_3_ receptor model, fragments were successively docked at each of the binding pockets. The top three hit fragments for the secondary site were fused to an arylpiperazine fragment with affinity for the primary site. The resulting compounds were docked in both the D_2_ and D_3_ receptors and were predicted to have high affinity for each receptor. Radioligand-binding assays showed that the *in vitro* binding affinity, but not the selectivity, of the compounds could be successfully predicted *in silico*. The binding assay was only reflective of orthosteric site binding, where the radioactive ligand can bind and be displaced (45). Alternatively, one binding pocket can be used to anchor fragment building toward the other pocket. To create selective lead compounds, a naturally derived fragment was docked in the allosteric pocket of the matrix metalloproteinase 13 (MMP-13) enzyme, then elongated until the molecule simultaneously occupied the orthosteric pocket (46).

While it may be logical to design inhibitors working through allosteric sites, the allosteric site may be undefined, as with the A_2A_ adenosine receptor. In this case, the hindrance of relying only on the orthosteric site for screening was mitigated by the NMR screen (40). Site-directed mutagenesis (SDM) is another complementary tool for defining allosteric sites. Such a site was first hinted at for the serotonin transporter (SERT) protein using SDM. Using the tentative allosteric SERT site to screen for non-competitive inhibitors yielded the novel SERT modulator ATM-7, displaying nanomolar affinity. Mutagenesis of the allosteric binding site residues predicted to interact with ATM-7 confirmed that SERT affinity for the allosteric ligand was lost (47). Computational approaches to discovering allosteric sites and the drugs that modulate receptor function via these sites will be key to developing efficient (and potentially selective) CNS drugs.

The β-amyloid cleavage enzyme BACE-1 and acetylcholinesterase (AChE) were recently studied using a group-based QSAR approach to designing fragments (48). Initially, ligand-based approach predicts new compounds by comparing functional groups of known inhibitors; structure-based screening was next applied. QSAR focused on compounds derived from 1,4-dihydropyridine was analyzed to predict protein interactions for different functional groups. The compounds were split into four pools based on a common functional group and were then docked into a crystal structure-based model. Molecules were initially screened using the BACE-1 model, followed by a secondary screen with the AChE model. This method produced leads that had dual functionality for both BACE-1 and AChE, representing a possible approach for Alzheimer’s drug design (48).

## Role of Medicinal Chemistry

Identifying legitimate candidate lead compounds from a library has always been a challenge with VS. Dahlin and Walters (35) argue that a majority of compounds being screened are “artifacts” or “promiscuous bioactive molecules,” which do not make for good lead targets. To address this problem, the authors recommend a triage approach to drug discovery involving a collaboration of medicinal chemists, biologists, and purification experts from the beginning of the drug design process. This approach of identifying targets for optimization allows for an exchange of ideas with experts. Medicinal chemists are placed in a pivotal role, defining which molecules are actually synthesizable. This approach is widely adopted by the pharmaceutical industry for the development of novel targets (35). Useful perspectives on how medicinal chemists approach drug design would include the reactions commonly in their toolboxes (49), and how they exploit molecular interactions (14). Incorporation of medicinal chemist expertise into computational methods is improving the drug design process.

### Application of “medicinal chemistry rules”

A first effort in this direction was Drug Guru™, a web-based program that applies medicinal chemistry rules to a starting fragment (50). Traditional medicinal chemistry approaches for creating new compounds employ (1) bioisoteric replacements: structural changes that retain similar properties or (2) non-classical replacements: more radical attempts to achieve a dramatic impact on a desired property. Initially, Drug Guru contained 187 reaction rules concerning functional group transformations and framework modifications. Results could then be manually inspected by the synthetic chemist. Drug Guru does not filter molecules, allowing the researcher to see all the options. The program compiles not only results expected by the medicinal chemist but also options that might not be normally considered. Drug Guru allows for multiple program cycles, further diversifying the product pool. Some newer software programs utilize the reaction rules from Drug Guru while adding the option to filter and dock newly generated compounds.

Segall et al. (51) described an additional medicinal chemistry transformation method. Drawing from the medicinal chemistry literature, 206 transformation reactions were divided into seven groups: ring addition, modification, and removal, functional group addition, linker modification, atom removal, and terminal group exchange. A reaction transformation language (SMIRKS) (52) was used to encode the transformations, and the StarDrop software platform (53) was used to apply the modification to a parent (starting) fragment using criteria supplied by the researchers. The user controls growth of the molecule in that reaction subsets may be selected, and regions of the parent molecule can be preserved. Using as parent molecule, the lead compound that led to the SERT inhibitor and antidepressant/analgesic duloxetine (Cymbalta™), QSAR models of absorption, distribution, metabolism, and elimination (ADME) properties and predicted SERT Ki values were used to predict hit compound pharmacological activity. Repeated application of this method was able to create an exponential number of diverse compounds. A set of 1500 compounds generated from 400 molecules was randomly assessed for quality by medicinal chemists, 94% of which were found to be acceptable (51).

AutoGrow is another software package that incorporates medicinal chemistry knowledge into ligand design (54). AutoGrow modifies the initial fragment through “mutations” that replace or combine reactive groups and “crossover” reactions that compare overlapping fragments with similar structures. A selection process compares the products regarding drug-like properties. Hit compounds are subsequently docked using AutoDock Vina (55) and scored by predicted binding affinity. The top-scoring compounds are selected for successive generations of modifications. The latest version of AutoGrow attempts to create compounds that are more easily synthesized (56).

BioSolveIT Inc. (Bellevue, WA, USA) has developed useful suites for FBDD and its support. The ReCore module modifies hit compounds by replacing their “core” (chemical scaffold) (57). Fragments used to replace the core are generated in 3D, a vector-based scheme is used to cut and replace the fragments, and the resulting structures are scored using the FlexX docking program. Filters can be applied to sort structures by size of fragment used or various geometic properties, such as torsion angles. The Feature Trees (FTrees) module explores chemical spaces with fragment hopping and using overlapping fragments to create a composite ligand structure (58). The starting fragment is modified with linker fragments that share similar functional groups; these are overlapped and the new fragment is grafted onto the starting fragment. The resulting structures should maximize the chemical space within the receptor (59). FTrees interfaces with PipelinePilot (SciTegic) and Molecular Operating Environment (MOE; Chemical Computing Group) software; the latter can be used to cluster FTrees results by topology (57). The fragment space extension module FTrees-FS allow a search of 10^18^ compounds in 5 min. BioSolveIT’s structure-based SeeSAR module provides receptor-binding affinity ­estimates that indicate atomic contributions within the compound, ranking hit compounds (also against known ligands, if desirable) even while they are being modified *in silico*.

Chemical Computing Group (Montreal, QC, USA) has recently added the MedChem Transformations (MCT) feature to their MOE software suite. Fragment growth or novel ligand scaffold building occurs within the ligand-binding pocket of the three-dimensional receptor target, providing advantages over two-dimensional approaches, such as QSAR. This *de novo* process, based on Drug Guru concepts, creates novel ligands using over 170 transformation rules. MCT begins with a starting fragment ligand in the receptor-binding pocket. Transformation rules are applied to discrete portions of the fragment using a match-and-replace algorithm. Once a match is made, the corresponding atoms are replaced and the unaffected portion of the ligand is added back to the newly created molecule. The transformation takes place in a 2D environment, and either minimization or 3D embedding generates 3D coordinates for the molecule. After filtering based upon molecular weight, molecular interactions, toxicity, solubility, or other chemical attributes, the molecule is assigned a synthesis feasibility score. The molecules can be refined using force fields and scored using the MOE docking program. MCT may generate bioisosteric (addition of a functional group with similar electrochemical properties) or homologation (addition of a repeating unit for functional group) transformations. The smaller and simpler the starting fragment, the greater number of iterations needed to add enough functional groups to create a molecule the size of a binding pocket-filling drug. In some cases, multiple iterations are necessary for the desired functional groups to be placed at the correct carbon positions. With these outcomes, large numbers of compounds are generated. Even with clustering or fingerprinting as a sorting mechanism, more results are obtained than an individual can effectively inspect.

### Application of medchem transformations

**Figure 5:**
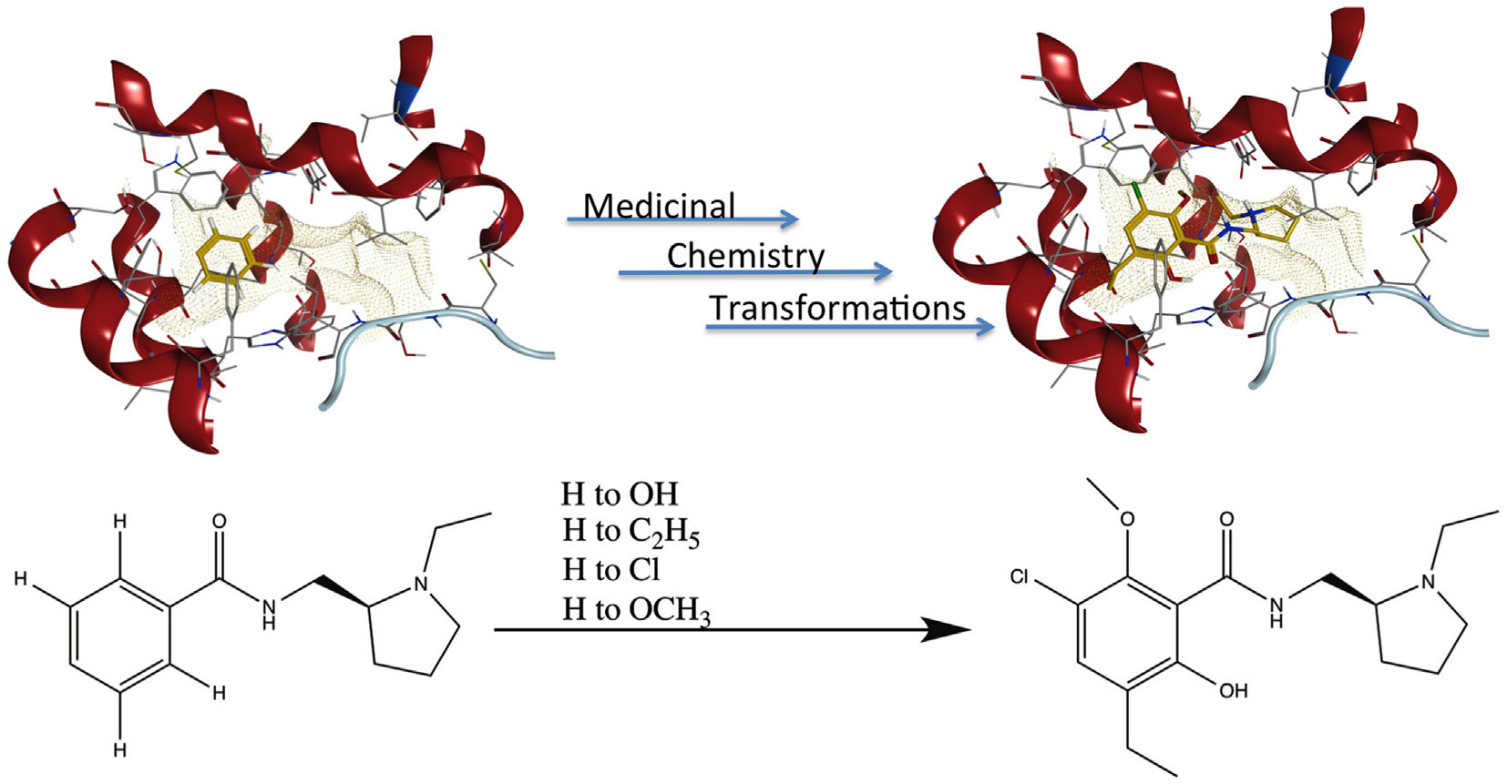
**Fragment growing in MedChem Transformations**. Upper panel: in theory, a starting fragment as simple as a benzene ring (yellow) can be grown into the D2/D3 antagonist eticlopride (yellow with atomtype-colored heteroatoms) within the D3 receptor (red) binding pocket with multiple MCT iterations. Lower panel: MCT chemistry in regenerating four substituent groups removed from eticlopride.

Publication of the x-ray coordinates of the dopamine D3 receptor complexed with the D2/D3 antagonist eticlopride (13) provided an opportunity to study FBDD methodology in a relatively controlled system. Eticlopride’s antagonist status meant that the usual (complicating) GPCR conformational changes upon agonist binding would be absent. The presence of this ligand in the crystal structure serves to define well the orthosteric antagonist pocket. In theory, MCT should be able to generate eticlopride within this pocket from a fragment as elementary as a benzene ring (Figure 5, upper panel). As a starting point in using this software, MCT was tested for its ability to rebuild eticlopride when a fragment lacking as many as three of the drug’s substituents was employed as the parent fragment (Figure 5, lower panel).

MedChem Transformations readily rebuilt eticlopride when single functional groups, such as the phenyl ring’s chloro or hydroxyl substituents, were first replaced with hydrogen atoms. This involved choosing a small set of transformations from the GROWTHRXN database (included in the software suite), and selecting the hydrogen that replaced the deleted functional group. Simultaneous regeneration of both the chloro and hydroxyl substituents was more challenging, requiring three synthesis iterations that yielded eticlopride and 63 other products. Additionally and simultaneously regenerating a third substituent (the ethyl moiety) required six iterations to obtain eticlopride among >2000 product compounds (Table 3). It should be noted that MCT was primarily designed to produce bioisosteres of a compound, as opposed to building a molecule from a single functional group. Furthermore, the smaller the starting fragment used, the greater number of iterations needed to generate a drug-like compound. This number of iterations is unknown beforehand, as is how long the program will need to run to produce the desired results.

**Table 3:** **MCT regeneration of eticlopride**.

Eticlopride	Substituent modified	Iterations	Number of products formed
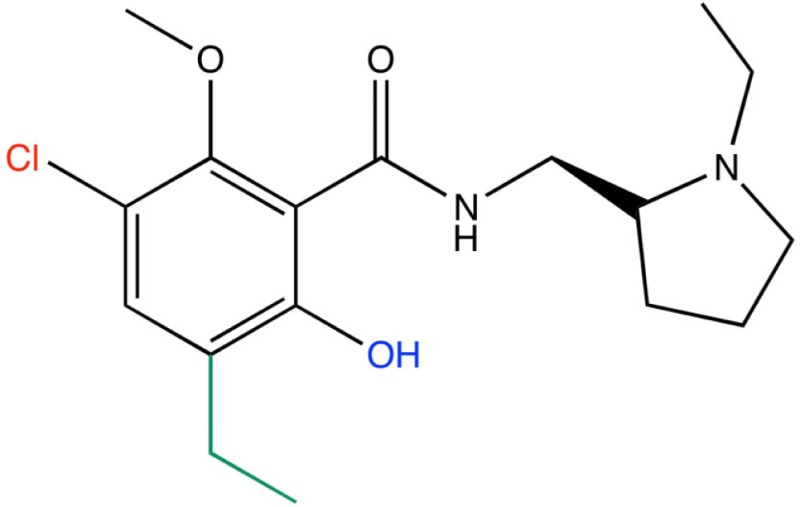	–Cl	2	13
	–OH	1	2
	–Cl, –OH	4	89
	–OH, –Cl, –Ethyl	6	2483

Interestingly, separate work recently successfully demonstrated the dopamine D3 receptor as a novel system to create ligands through FBDD. Utilizing the D3 receptor crystal structure (13) and a D2 receptor homology model, compounds with greater selectivity for the D3 receptor were computationally predicted using docking via the Glide 5.9 software, and validated *in vitro* (45).

## Conclusion

The exorbitant cost of drug development is a driving force behind changes in the pharmaceutical industry. R&D has become a target of cutbacks and a victim to outsourcing. The continued development of *in silico* methodology enhances the speed and cost effectiveness of drug discovery. Rapid advances in our understanding of molecular mechanisms of action underlying depression/anxiety, schizophrenia and bipolar disorder, substance abuse, and Alzheimer’s and Parkinson’s diseases are providing new CNS target proteins for pharmacotherapeutic intervention. The addition of FBDD to structure-based VS should increase the structural variety of hit-to-lead compounds.

Because the tools required for *in silico* discovery are accessible and affordable to an academic researcher, drug discovery now extends beyond the pharmaceutical industry. Techniques, such as the multitarget growing strategy, the sequential docking method, and group-based QSAR, allow development of fragments into lead molecules. MCT is a FBDD method that creates novel ligands with a high degree of synthesizability. It is by the exploration of diverse transformations that truly unique lead molecules can be formed, but such computations are not without their disadvantages. The task of computationally sampling an essentially limitless number of structures is time consuming and resource demanding. Another challenge is sorting potential structures appropriately, which currently is subject to human judgment. Issues such as these will have to be addressed in the future. Overall, FBDD strategies provide diverse and useful tools that will lead to the development of medications that could not be predicted by conventional structure–activity relationship-based methods.

## Conflict of Interest Statement

The authors declare that the research was conducted in the absence of any commercial or financial relationships that could be construed as a potential conflict of interest.

## Funding

This work was supported by NIH grant DA027806.

## Footnotes

^1^www.rcsb.org

^2^http://www.ccdc.cam.ac.uk

## Abbreviations

ADME, absorption, distribution, metabolism, and elimination; FBDD, fragment-based drug design; GPCR, G protein-coupled receptor; LBDD, ligand-based drug discovery; MCSS, multiple-copy simultaneous search; MCT, MedChem Transformations; MOE, molecular operating environment; NMR, nuclear magnetic resonance; QSAR, quantitative structure–activity relationship; SBDD, structure-based drug discovery; SDM, site-directed mutagenesis; SERT, serotonin transporter protein; VHTS, virtual high-throughput screening; VS, virtual screening.
